# Groundwater Controls on Plant Community Structure and Species Specific Water Use Strategies in the Desert Oasis Ecotone of the Junggar Basin

**DOI:** 10.1002/ece3.73443

**Published:** 2026-04-07

**Authors:** Meifei Zhu, Youyan Zhang, Xianglian Wang, Jinhua Cheng, Chunying Lei, Zhengwei Han

**Affiliations:** ^1^ College of Soil and Water Conservation Beijing Forestry University Beijing China; ^2^ Institute of Ecological Conservation and Restoration Chinese Academy of Forestry Beijing China; ^3^ Xinjiang Jinghe Desert Ecosystem National Positional Observatory, Institute of Silviculture and Sand Control Xinjiang Forestry Academy of Sciences Urumqi China

**Keywords:** desert oasis transition zone, ground water, MixSIAR model, niche overlap, stable isotopes, water source

## Abstract

Desert oasis ecotones are highly sensitive, yet the mechanisms through which groundwater fluctuations regulate community structure via hydrological niche dynamics remain unclear. To address this gap, we investigated plant water use strategies and niche overlap along a natural groundwater gradient (4.8 m, 9.4 m, 11.8 m) by combining plant surveys with stable isotope analysis using MixSIAR and nicheROVER models. Species richness exhibited a unimodal response to groundwater depth, peaking at the intermediate level, whereas both shallow and deep groundwater conditions reduced richness due to salinity and water limitation, respectively. *Haloxylon ammodendron* displayed contrasting population structures, with strong regeneration under shallow groundwater but dominance of smaller, senescent individuals at deeper depths. Its water source shifted progressively from groundwater to unsaturated zone soil water as depth increased. *Calligonum mongolicum* primarily relied on shallow to mid depth soil water and exhibited moderate plasticity at intermediate depth. In contrast, *Tamarix chinensis* and *Ephedra intermedia* consistently utilized deep water sources. Niche overlap analysis revealed a nonlinear pattern: extreme water stress induced complete niche separation, moderate resource availability at the intermediate depth promoted temporary high overlap coexistence, and secondary stress coupled with competition at the shallow depth led to competitive niche sorting. Across the gradient, the dominant (*H. ammodendron*) and specialized ( *C. mongolicum* ) species maintained stable and differentiated ecological roles, respectively. Our findings establish a nonlinear framework linking groundwater depth, species strategies, and hydrological niche dynamics. This mechanism profoundly influences community stability, providing critical insights for predicting plant dynamics and guiding groundwater management in arid ecosystems.

## Graphical abstract

We investigated how groundwater fluctuations shape desert oasis ecotone communities via hydrological niche dynamics across a 4.8–11.8 m groundwater gradient. Species richness peaked at intermediate depths, as shallow zones faced salinity constraints and deep zones faced water limitation; plant water‐use strategies and population structures diverged sharply with depth. Niche overlap followed nonlinear patterns: full separation under extreme stress, high coexistence overlap at moderate resources, and competitive sorting in shallow zones. Our findings link groundwater depth, species strategies, and niche dynamics, providing insights for arid ecosystem management and community stability predictions.

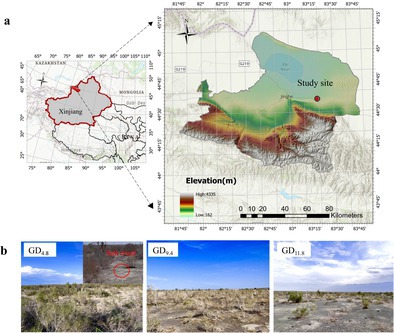

## 1 Introduction

The desert oasis ecotone, located at the interface between desert and oasis ecosystems, is one of the most ecologically sensitive zones in arid regions and serves as a critical buffer for combating desertification and maintaining regional ecological stability (Zhang et al. 2017). In this fragile boundary zone, xerophytic woody species such as *Haloxylon ammodendron* play irreplaceable ecological roles, not only in stabilizing sand dunes and maintaining soil structure, but also in supporting ecosystem functioning and promoting vegetation recovery (Ji et al. 2016). However, the growth and community dynamics of these species are highly dependent on water availability, which is constrained by sporadic precipitation and limited hydrological connectivity between soil and groundwater (Jiao et al. 2021; Luo et al. 2023). In arid environments where rainfall is scarce and uneven, groundwater becomes the key water source sustaining plant growth and community stability, with its stability, spatial accessibility, and temporal fluctuations determining vegetation persistence and ecosystem recovery capacity (Luo et al. 2023; Wang et al. 2023).

Groundwater depth dynamics are recognized as a major driver of community composition and population structure in arid zone vegetation (Soylu et al. 2014; Xu and Su 2019). An increase in groundwater depth may alleviate shallow soil salinization driven by saline groundwater capillary rise, but simultaneously increases the cost of water acquisition (Huang et al. 2020). Such hydrological changes, triggered by water table fluctuations, can reshape community structure by regulating the growth, regeneration, and competitive balance between dominant and subordinate species (Sommer and Froend 2014). For instance, *H. ammodendron* populations exhibit pronounced reductions in individual size and density under deeper groundwater conditions, reflecting their high sensitivity to water table fluctuations (Mu et al. 2024). Groundwater dynamics may also influence canopy cover, crown size, and biomass allocation of species, thereby altering population age structures and regeneration potential, with long‐term implications for community stability (Ahring and Steward 2012).

To cope with water limitation driven by hydrological constraints, desert plants have evolved multiple adaptive strategies to optimize water use (Cao et al. 2020; Wang et al. 2021). On the one hand, many woody xerophytes develop dimorphic root systems, where shallow roots rapidly absorb surface and shallow soil water, while deep taproots exploit relatively stable groundwater, enabling vertical partitioning of water sources (Dawson and Pate 1996; Pei et al. 2023; Zhao et al. 2021). This structural adaptation confers high plasticity in water uptake (Wu et al. 2022), allowing plants to flexibly adjust their strategies under different groundwater conditions and maintain ecological functions. On the other hand, in multispecies communities, plants often achieve hydrological niche differentiation to reduce direct competition (Priyadarshini et al. 2016; Zhao et al. 2021). By exploiting distinct soil layers or water sources across spatial or temporal scales, coexisting species achieve niche separation (Gu et al. 2025), which enhances community stability and productivity. Crucially, however, the diversity of these water use strategies is not limitless; rather, it is fundamentally constrained by the coordination and trade‐offs of plant functional traits (Muñoz‐Gálvez et al. 2025). These findings collectively indicate that groundwater not only regulates the water use strategies of individual species within their physiological limits, but also profoundly influences interspecific interactions within communities. Nevertheless, despite advances in understanding vertical partitioning and niche differentiation of water use in xerophytes (Fan et al. 2025; Tiemuerbieke et al. 2018), how groundwater fluctuations shape species‐specific strategies and hydrological niche allocation at both population and community levels remains poorly quantified, particularly in desert oasis ecotones where hydrological processes and human activities strongly interact (Qiu et al. 2023). Given this, studying along a natural groundwater depth gradient is the most effective way to resolve the nonlinear impacts of environmental stress on community structure and niche dynamics, as a single water level condition cannot fully reveal the complete range of species adaptation mechanisms and plasticity.

To accurately capture the complete regulatory effects of groundwater fluctuations on niche allocation and community composition, this study adopted a sampling strategy along a gradient of differing groundwater depths. To this end, we employed stable isotope techniques (δ^2^H and δ^18^O) as a key tool to trace plant water sources (Miguez‐Macho and Fan 2021). The isotopic composition of xylem water closely reflects the signature of the main water sources, and comparison with precipitation, soil water, and groundwater allows determination of plant water uptake depth and relative source contributions (Rothfuss and Javaux 2017; Wang et al. 2017). Moreover, isotope‐based mixing models such as MixSIAR can quantitatively partition multiple water sources, offering robust approaches to reveal interspecific water use differentiation and community niche patterns (Bello et al. 2019; Chen et al. 2020) Resolving these isotopic patterns provides a vital mechanistic basis for understanding how physiological trade‐offs constrain water use diversity in dryland ecosystems (Muñoz‐Gálvez et al. 2025). Integrating vegetation surveys with isotopic analysis thus enables a cross‐disciplinary hydrological and ecological perspective to explore how groundwater regulates community composition, population structure, and plant water use strategies, thereby providing a scientific basis for plant restoration and groundwater management in arid regions.

Based on this background, the present study focuses on the desert oasis ecotone of the Junggar Basin, northwestern China, to investigate how groundwater depth gradients influence community composition, population structure of *H. ammodendron*, and water use strategies of coexisting species. Specifically, our objectives were: (1) to evaluate the structural responses of *H. ammodendron* populations under different groundwater conditions; (2) to quantify species‐specific water use strategies of *H. ammodendron*, *Calligonum mongolicum*, *Tamarix chinensis* , and *Ephedra intermedia* using stable isotope data of soil water, groundwater, and xylem water; and (3) to examine patterns of hydrological niche separation and overlap among coexisting species along groundwater gradients. We hypothesized that: (1) along the groundwater depth gradient, *H. ammodendron* would exhibit flexible water source utilization, shifting toward deeper sources under increasing hydrological stress, accompanied by changes in population structure; and (2) coexistence between *H. ammodendron* and *C. mongolicum* would be stress dependent, with higher niche overlap under intermediate groundwater conditions and stronger niche differentiation under shallow (salinity related) and deep (water limited) stress. By addressing these questions, this study aims to contribute to a conceptual understanding of groundwater plant interactions in arid ecosystems and to provide scientific guidance for predicting plant responses to water scarcity and for informing conservation and management strategies in fragile ecotones.

## 2 Materials and Methods

### 2.1 Study Site

The study was carried out in Jinghe County, Bortala Mongol Autonomous Prefecture, Xinjiang, China, located at the junction between the northern foothills of the Tianshan Mountains and the southern edge of the Junggar Basin (81°44′~83°45′ E, 40°00′~45°10′ N). The area's geomorphology is characterized by a premountain alluvial floodplain inclined plain, with terrain gently sloping from south to north, featuring a flat and open landscape. The region experiences a typical north temperate arid desert continental climate, with average temperatures of −15°C in January and 26°C in July. Extreme temperatures range from a maximum of 44°C to a minimum of −33°C. Annual precipitation is limited, averaging only 104 mm, while annual evaporation is around 1498 mm.

The dominant native plants in the area include *H. ammodendron*, *C. mongolicum* , *E*., *T. chinensis*. After being degraded in the 1980s, local government initiated aerial and horseback seeding to carry out replanting. Subsequently, plant recovery has been maintained solely through natural regeneration. Consequently, the current *H. ammodendron* populations represent a mixed demographic, consisting of both the surviving aging individuals from the initial 1980s artificial seeding and their naturally regenerated descendants. However, in recent years, these restored communities have suffered degradation due to falling water tables and the resumption of grazing activities (Yin et al. 2022).

### 2.2 Experimental Design

**FIGURE 1:**
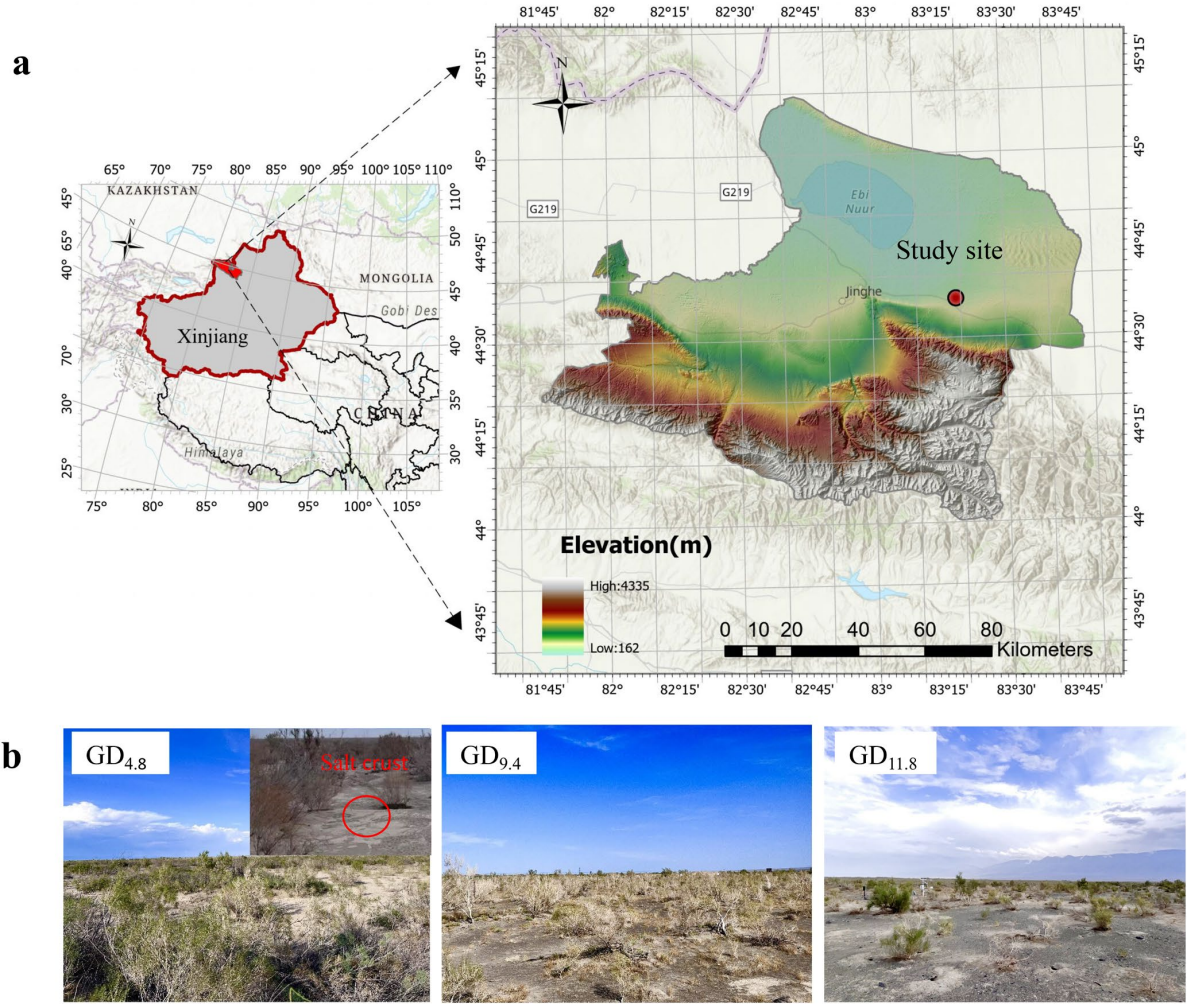
Geographical location and ecological landscape of the study area. (a) Location of study area; (b) Representative photographs of sample plots under different groundwater depths (4.8 m, 9.4 m, and 11.8 m).

Three sites were established along a north south gradient on flat terrain, perpendicular to the Kornu Mountains, a branch of the Tianshan Range. These sites, designated GD_11.8_, GD_9.4_, and GD_4.8_, represent three community types (Figure 1) with mean groundwater depths of 11.8 m, 9.4 m, and 4.8 m, respectively. The groundwater depth data were derived as multiyear averages from monitoring wells at each site. Annual water level fluctuations, which were less than 0.7 m. All plots (Table 1) were situated within a 10 km distance of one another on gentle slopes. For each groundwater depth, three 20 m × 20 m quadrats were randomly established, each containing naturally regenerated *H. ammodendron* communities. Within each quadrat, the basal diameter, height, and crown width of every *H. ammodendron* individual were recorded. All other woody and herbaceous species present in the quadrats were identified and counted. Species abundance was defined as the total number of individuals of a given species per quadrat, and relative abundance was calculated as the proportion of individuals of that species relative to the total number of individuals in the quadrat.

**TABLE 1:** The basic overview of the sampling point.

Plot	Elevation (m)	Main plant types
GD_11.8_	300	*H. ammodendron, C. mongolicum*
GD_9.4_	262	*H. ammodendron, C. mongolicum, E. intermedia*
GD_4.8_	240	*H. ammodendron, C. mongolicum, T. chinensis*

### 2.3 Field Sampling

Plant and soil samples were collected monthly from August to September 2023. On each sampling date, 3–5 healthy individuals of *H. ammodendron*, *C. mongolicum* , *E. intermedia* , and *T. chinensis* were selected. Five woody xylem samples were taken from each individual after removing all green tissues to prevent isotope fractionation. The samples were immediately sealed in screw‐cap glass bottles with parafilm and stored at −20°C until water extraction. In total, 102 xylem samples were collected.

The sampling period (August–September) corresponds to the late growing season, when vegetative and reproductive development of dominant desert shrubs is relatively stable and plant water use strategies are less influenced by rapid phenological shifts (Chen and Zuo 2019). In addition, precipitation during this period is typically low and episodic in the study region. Although rainfall samples were included in the isotope analysis, the limited precipitation input reduces short‐term disturbances to soil and groundwater isotopic composition, facilitating clearer identification of plant water sources. This period is therefore commonly considered suitable for identifying relatively stable plant water use strategies in arid ecosystems.

Soil samples were collected simultaneously using a handheld auger to a depth of 300 cm around each sampled plant. Soil was sampled at 20 cm intervals within 0–120 cm and 50 cm intervals within 150–300 cm, yielding 10 depth layers. Three replicates were collected at each depth, and samples from the same depth within each plot were composited, resulting in 180 soil isotope samples. A portion of each sample was sealed and stored at −20°C for water extraction, while the remaining soil was oven‐dried at 105°C to determine soil water content (SWC). The formula for calculating SWC is as follows.

(1) $$\mathrm{SWC}=\frac{s_{f}-s_{d}}{s_{d}}$$

where *S*_f_ and *S*_d_ represent the weights of the fresh and dried soil (g), respectively.

Rainwater was collected using homemade rainwater collectors, which consisted of an open bottle with a funnel attached to its opening. A ping‐pong ball was placed on the funnel to prevent water evaporation. After each rainfall, the collected rainwater was transferred into screw‐cap glass bottles, labeled with the corresponding sampling time, sealed with parafilm, and stored at −4°C for future isotope analysis. A total of 21 rainwater samples were obtained from seven separate rainfall events during the study.

A dedicated groundwater monitoring well was established at each of the three study sites (GD_11.8_, GD_9.4_, GD_4.8_) to characterize site specific hydrological conditions. Groundwater sampling was conducted once per month during August and September 2023. During each sampling event, three parallel samples were collected from each well to ensure analytical reliability. Consequently, the total number of groundwater samples was 18. All water samples were subsequently analyzed for hydrogen (δ^2^H) and oxygen (δ^18^O) stable isotopes.

### 2.4 Determination of the Stable Isotopic Composition

Water extraction from plant and soil samples was conducted using an automated vacuum condensation‐extraction system (LI‐2100, LiCa United, Beijing, China) with an extraction duration of 4 h. Thereafter, the extracted xylem water, soil water, rainfall, and groundwater were filtered through 13 mm × 0.22 μm organic phase syringe filters to remove organic contaminants (Fan et al. 2025), and the filtered water samples were then transferred into 2 mL glass vials. The ^2^H and ^18^O isotope compositions of the samples were subsequently determined using a liquid water isotope analyzer (DTL‐100, LGR, Lawrenceville, USA). Analytical precision was ±0.1‰ for δ ^2^H and ±0.3‰ for δ ^18^O. To account for potential spectral interference from organic compounds, correction curves were applied to all samples. To this end, correction curves were utilized to eliminate the effects of organic contaminants present in the water samples. The analytically derived ^2^H and ^18^O were expressed as relative Vienna Standard Mean Ocean Water (VSMOW) parts per thousand (‰):

(2) $$\begin{array}{c} \mathrm{\delta X}\left(‰\right)=\frac{R_{\text{sample}}-R_{\text{vsmow}}}{R_{\text{vsmow}}}\times 1000 \end{array}$$

where δ is the deviation of the isotope ratio of a sample relative to that of the VSMOW. *X* denotes ^2^H and ^18^O, *R*_sample_ represents ^2^H/^1^H and ^18^O/^16^O of the samples, and *R*_vsmow_ refers to ^2^H/^1^H and ^18^O/^16^O of the VSMOW.

Deuterium excess (d‐excess) was calculated as *d* = δ^2^H—8 × δ^18^O (Dansgaard 1964) to assess the degree of secondary evaporation.

### 2.5 Quantifying the Water Source

The MixSIAR model, a Bayesian stable isotope mixing model, was employed to quantify the proportional contributions of multiple sources to a mixture based on biotracer data (Moore and Semmens 2008). In studies apportioning water sources for woody plants, MixSIAR has demonstrated superior performance compared to other Bayesian mixing models, such as SIAR and MixSIR (Wang et al. 2019). Isotope values (δ^2^H and δ^18^O) of water xylem were used as the mixture data. The source data comprised the mean and standard deviation of δ^2^H and δ^18^O values from the following sources: rainfall, shallow soil water (0–80 cm), intermediate soil water (80–150 cm), deep soil water (150–200 cm), and groundwater. The soil profile was divided into three layers based on both isotopic and hydrological patterns observed in this study. The shallow layer (0–80 cm) showed significantly enriched isotope values (δ^2^H and δ^18^O; *p* < 0.05; Figure 4b), indicating strong evaporative fractionation. In contrast, soil water content in the deep layer (> 150 cm) exhibited a significant response to groundwater depth (Figure 5), suggesting hydraulic connectivity with groundwater and relatively stable storage conditions. The intermediate layer (80–150 cm) therefore represents a transitional zone influenced by both shallow evaporation and deeper water sources. Accordingly, the soil water sources were categorized into shallow (evaporation layer), intermediate (transition layer), and deep (capillary or relatively stable layer) for the MixSIAR analysis (Gu et al. 2025). Fractionation was assumed to be “0”, indicating no isotopic fractionation by default. The Markov Chain Monte Carlo (MCMC) procedure was configured with a prolonged “burn‐in” period, and the error structure was set to “Residual Only”. Convergence was assessed using Gelman‐Rubin and Geweke diagnostics. If convergence was not reached, the burn‐in period was extended until it was confirmed. The final output results were presented as averages.

### 2.6 Quantification of Hydrological Niche

Hydrological niche overlap and separation were quantified using the ‘nicheROVER’ R package, based on δ^2^H and δ^18^O values of plant xylem water. This Bayesian method calculates the probability that an individual from species A is found within the 95% niche region of species B, and vice versa, yielding asymmetric pairwise probabilities (Swanson et al. 2015). The analysis utilized 1000 posterior samples per species to define the niche regions and estimate overlap probabilities, with lower values indicating greater niche separation.

(3) $$\begin{array}{c} O\left(\begin{array}{c} A\\ B \end{array}\right)=\mathrm{Pr}\left(X_{A}\in N_{R}\left(B\right)\right) \end{array}$$

$O\left(\begin{array}{c} A\\ B \end{array}\right)$ denotes the niche overlap of species A onto species B, representing the probability that an individual from species A occurs within the niche region of species B. Pr stands for “Probability”, used to quantify the likelihood of an event occurring. *X*_*A*_ represents a randomly selected individual from species A. *N*_R_(B) denotes the niche region of species B, the range occupied by species B in the environmental space.

### 2.7 Data Analysis

Statistical analyses were performed using IBM SPSS Statistics 21 and R software. For continuous variable relationships, we applied linear regression to assess the association between basal diameter and tree height, and quadratic regression to evaluate the unimodal relationship between species richness and groundwater depth. To compare group differences, one‐way ANOVA (followed by Tukey's post hoc test) was used for normally distributed data, while non parametric tests were applied when normality assumptions were not met.

## 3 Results

### 3.1 Plant Community Composition and Population Structure Responses to Groundwater Depth Gradients

#### 3.1.1 Plant Community Responses to Groundwater Depth Gradients

Plant surveys demonstrated pronounced shifts in community composition along the groundwater depth gradient (Table 2). The relationship between species richness and groundwater depth was described by a significant unimodal pattern (quadratic regression: *β* = −0.12, *p* = 0.03), with the highest mean richness observed at the intermediate depth of 9.4 m. *H. ammodendron* (Ha) consistently dominated across all depths, maintaining a relative abundance above 60%, thereby reinforcing its status as a dominant species in this desert ecosystem. Species specific responses, however, varied substantially. The relative abundance of *C. mongolicum* (Cm) declined sharply from 30.71% to 12.73% with decreasing groundwater depth, indicating sensitivity to shallower conditions. In contrast, *T. chinensis* (Tc) increased by 25.14%, reflecting stronger adaptability to shallow groundwater. By comparison, both *H. ammodendron* and *E. intermedia* (Ei) exhibited nonlinear relative abundance trends, reaching their peak values under intermediate groundwater depth conditions.

**TABLE 2:** Species composition and relative abundance of communities under different groundwater gradients.

Plant species	Percentage (%)		
	GD_11.8_	GD_9.4_	GD_4.8_
*H. ammodendron*	65.57 ± 10.35a	67.35 ± 2.94a	61.33 ± 6.09a
*C. mongolicum*	30.71 ± 8.21Ab	23.70 ± 4.03ABb	12.73 ± 6.64Bc
*E. intermedia*	0.53 ± 0.92c	3.08 ± 2.56c	0.80 ± 0.48d
*T. chinensis*	0Ac	3.06 ± 2.20Ac	25.14 ± 6.25Bb
*Xylosalsola arbuscula*	1.06 ± 1.82c	2.04 ± 0.68c	0d
*Reaumuria songarica*	2.12 ± 1.38c	0.77 ± 1.33c	0d

*Note:* Different uppercase and lowercase letters denote significant differences (*p* < 0.05) in species relative abundance among different sites and within the same site, respectively.

#### 3.1.2 Population Structure Dynamics of *H. ammodendron* Along Groundwater Depth Gradients

**FIGURE 2:**
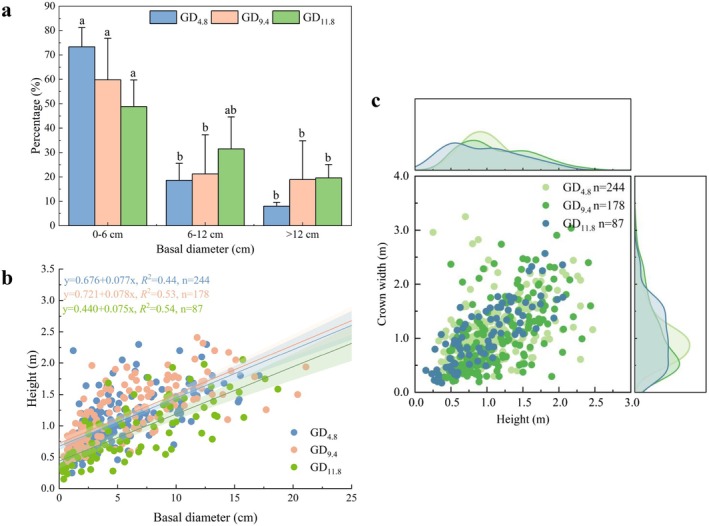
Characteristics of basal diameter (a), basal diameter and tree height (b), height and crown width of *H. ammodendron* at different groundwater depths in the desert oasis transition zone of the Junggar Basin (c). Different lowercase letters indicate significant differences in the proportion of different basal diameters at different depths of groundwater (*p* < 0.05).

Population structure analysis of *H. ammodendron* further revealed a significant negative correlation between basal diameter and individual abundance (*r* = −0.68, *p* < 0.05). Small diameter individuals (basal diameter < 6 cm) accounted for the majority of the population (Figure 2a), while large diameter individuals were relatively rare. With increasing groundwater depth, the proportion of large diameter individuals increased; however, these trees exhibited reduced height, forming a dwarfed, senescent morphology. This growth constraint was quantitatively confirmed by the lowest slope (0.075) of the height to basal diameter relationship at the GD_11.8_ site (Figure 2b). Crown width distributions also varied with groundwater depth (Figure 2c), shifting from a unimodal pattern under shallow and intermediate depths to a broad plateau pattern concentrated in the 0.5–1.8 m range under deeper water tables. Collectively, shallower groundwater was associated with enhanced crown expansion, greater population density, and a higher proportion of young individuals. These findings underscore the critical regulatory role of groundwater depth in mediating the trade off between vegetative growth and individual maturation in *H. ammodendron* populations.

### 3.2 Isotopic Characteristics of Water Sources and Plant Xylem Across Groundwater Depths

#### 3.2.1 Isotopic Signatures of Precipitation, Soil Water, and Groundwater

**FIGURE 3:**
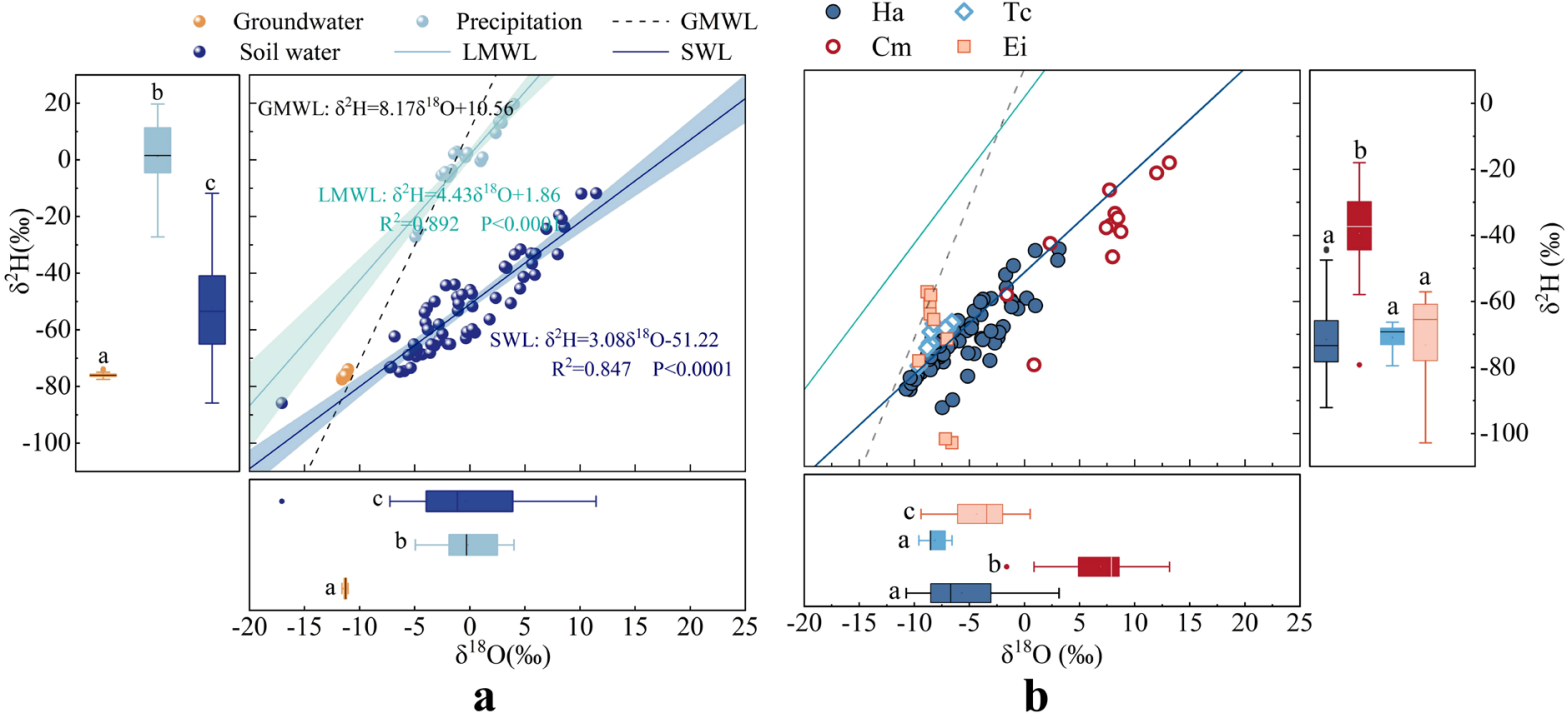
The isotopic compositions of hydrogen (δ^2^H) and oxygen (δ^18^O) in potential water sources (a), and the isotopic compositions of water in the xylem of various plant types (b). LMWL refers to the Local Meteoric Water Line, SWL denotes the Soil Water Evaporation Line, GWWL stands for the Global Meteoric Water Line. Ha stands for *H. ammodendron*, Cm for *C. mongolicum* , Ei for *E. intermedia* , and Tc for *T. chinensis* .

A linear regression between precipitation δ^18^O and δ^2^H during the study period yielded the local meteoric water line (LMWL) (Figure 3a):δ^2^H = 4.43δ^18^O + 1.86 (*n* = 21, *R* ^2^ = 0.89). The LMWL exhibited a relatively low slope (4.43) and intercept (1.86), deviating markedly from the global meteoric water line (GMWL). This pattern likely reflects strong sub‐cloud secondary evaporation, which is characteristic of arid summer climates (Liu et al. 2009). During the sampling period, precipitation events were generally small (all < 9 mm). Raindrops from such light rainfall events are susceptible to kinetic fractionation while falling through warm and dry unsaturated air, leading to isotopic enrichment and a reduced regression slope. Under such evaporative conditions, δ^2^H–δ^18^O relationships commonly approach the slope range typical of evaporation lines (4–6), which are widely observed in evaporating surface waters and arid environments (Vystavna et al. 2021). The soil water line (SWL) was expressed as: δ^2^H = 3.08δ^18^O—51.22 (*n* = 180, *R* ^2^ = 0.85). Most soil water isotopic data points plotted to the right of the precipitation line, indicating that soil water was primarily derived from precipitation but experienced varying degrees of evaporative enrichment across the sampling sites.

The δ^2^H values of the three potential water sources ranged from −77.51‰ to 19.71‰, and δ^18^O values ranged from −11.59‰ to 11.46‰, showing clear stratification (Figure 3a). Groundwater exhibited the most depleted signatures (δ^2^H: −77.51‰ to −73.86‰, *n* = 18), followed by soil water (δ^2^H: −73.42‰ to −11.95‰), while precipitation displayed the most enriched values (δ^2^H: −5.97‰ to 9.47‰). Overall, both δ^2^H and δ^18^O values followed a consistent hierarchy among sources: groundwater < soil water < precipitation.

#### 3.2.2 Xylem Water Isotopes and Inter Species Variations

Xylem water isotopic composition reflected plant water use patterns (Figure 3b). The δ^2^H of xylem water ranged from −102.85‰ to −17.98‰, and δ^18^O from −10.73‰ to 13.15‰. The isotopic signatures largely clustered along the SWL and GWWL, indicating that isotopic fractionation during root uptake and transport was negligible.

A one‐way ANOVA on δ^2^H values of *H. ammodendron* xylem water revealed no significant differences among the sites with different groundwater depths (GD_4.8_, GD_9.4_, GD_11.8_) (*p* > 0.05). However, δ^18^O values at GD_11.8_ were significantly higher than those at GD_4.8_ and GD_9.4_ (*p* < 0.001). Inter species comparisons revealed that *C. mongolicum* exhibited significantly higher δ^2^H values (−39.43‰) than *T. chinensis* (−70.92‰), *H. ammodendron* (−71.57‰), and *E. intermedia* (−73.24‰) (*p* < 0.001). Similarly, *C. mongolicum* had markedly enriched δ^18^O values (6.92‰) compared with *E. intermedia* (−4.32‰), *H. ammodendron* (−5.70‰), and *T. chinensis* (−8.13‰) (*p* < 0.001). These results highlight clear interspecific differences in water use strategies.

#### 3.2.3 Isotopic Profiles and Soil Water Content Across Soil Depths

**FIGURE 4:**
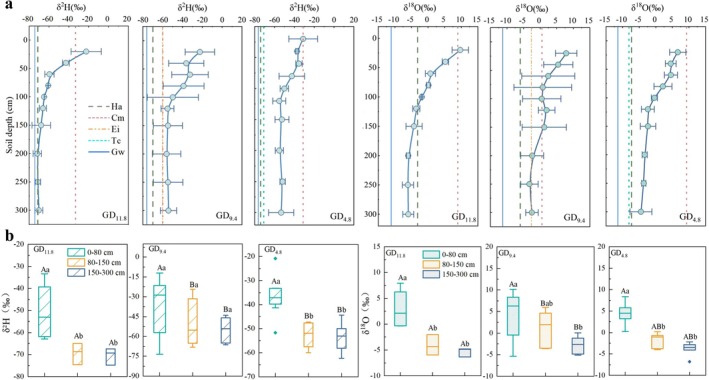
(a) Vertical distribution of soil water isotopes at different groundwater depths. The dashed line represents the isotopic composition of plant stem water, while the blue straight line represents the isotopic composition of groundwater. (b) Stratified distribution of soil water hydrogen and oxygen isotopes at different groundwater depths. Lowercase letters denote significant differences among soil layers within a depth, and uppercase letters denote significant differences among depths within a layer (*p* < 0.05).

The isotopic composition of soil water varied significantly along the groundwater depth gradient. For example, at GD_11.8_ (δ^2^H = −59.86‰, δ^18^O = −1.91‰), mean isotopic values were markedly more depleted compared with GD_4.8_ (δ^2^H = −46.50‰, δ^18^O = 0.11‰) and GD_9.4_ (δ^2^H = −46.46‰, δ^18^O = 1.21‰). As shown in Figure 4a, soil water isotopes generally decreased with increasing depth, forming a clear vertical stratification pattern. Except at the intermediate groundwater depth (Figure 4b), the upper soil layer (0–80 cm) in all sites exhibited significantly enriched δ^2^H and δ^18^O values (*p* < 0.05), reflecting stronger evaporative enrichment due to atmospheric exposure (Bowen et al. 2018). Except for the shallow layer (0–80 cm) where no differences were found, isotopic values in mid‐to‐deep soil layers were significantly lower at the shallow groundwater depth than at the deep groundwater depth (*p* < 0.05).

**FIGURE 5:**
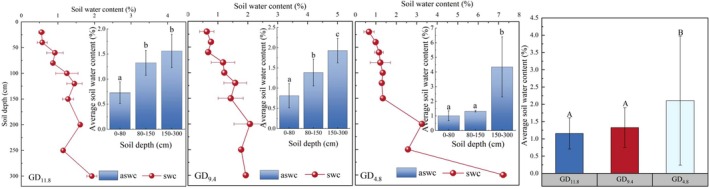
Vertical distribution of soil water content at different groundwater depths. Lowercase letters indicate significant differences among soil layers within the same groundwater depth, while uppercase letters indicate significant differences among groundwater depths (*p* < 0.05).

Soil water content measurements indicated that groundwater proximity had a significant regulatory effect on soil water availability (Figure 5). Soil water content ranged from 0.55% to 7.22%, with the mean value at GD_4.8_ (2.11%) being significantly higher than those at GD_11.8_ (1.16%) and GD_9.4_ (1.21%) (*p* < 0.05). Overall, soil water content increased with depth, with the steepest rise occurring near the saturated zone, highlighting the critical role of groundwater in recharging deep soil water. Across different groundwater depths, soil water content at the same depth generally showed no significant differences (*p* > 0.05), except in the 150–300 cm layer, where significant variation was observed. This layer is closer to the zone influenced by groundwater through capillary rise, and therefore its moisture conditions are more sensitive to groundwater depth. In contrast, shallow soil layers are primarily controlled by limited precipitation and strong evaporation, resulting in relatively uniform soil moisture across sites.

### 3.3 Species Specific Water Use Strategies and Niche Partitioning Along Groundwater Gradients

#### 3.3.1 Species Specific Water Use Strategies Along Groundwater Gradients

**FIGURE 6:**
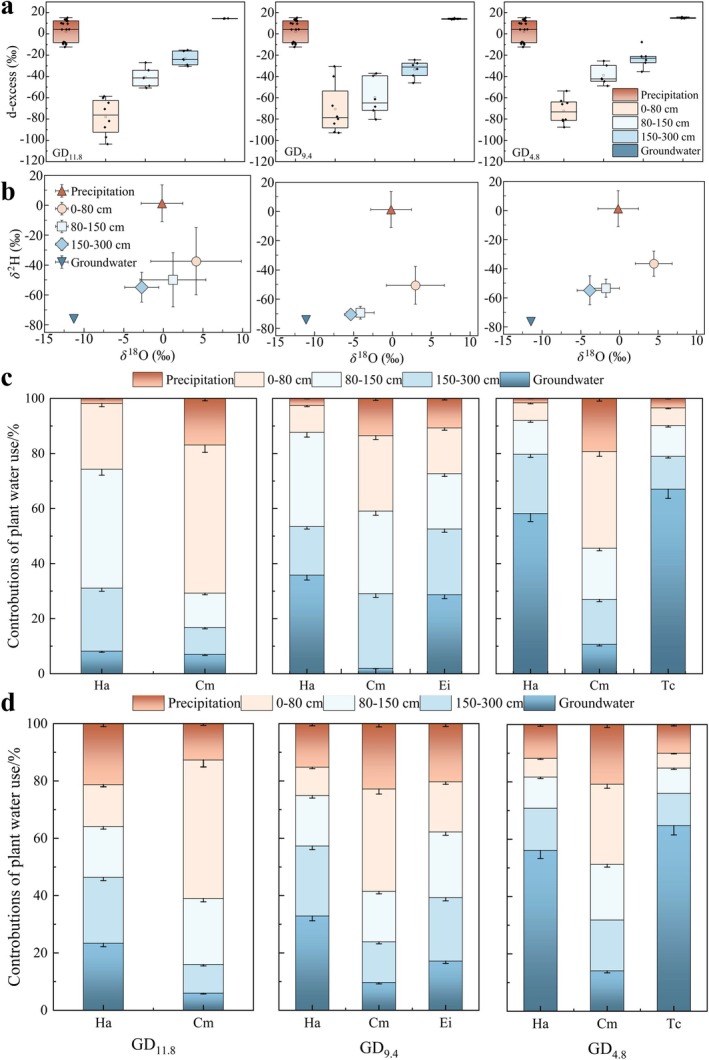
(a) d‐excess of different water sources under varying groundwater depth gradients. (b) Mean isotopic composition (δ^2^H vs. δ^18^O) of potential water sources at three groundwater depth sites. (c) Contributions of potential water sources calculated using both H and O isotopes. (d) Contributions of potential water sources calculated using only O isotopes.

To address potential uncertainties associated with hydrogen isotope fractionation in dryland plants (Ellsworth and Williams 2007; Yang et al. 2026; Zhao, Liu, et al. 2024), we repeated the MixSIAR estimations using δ^18^O data only (Figure 6d) and compared them with the dual‐isotope results (Figure 6c). The overall patterns were broadly consistent between the two approaches (*R* ^2^ = 0.67) (Figure S1), indicating that the main conclusions were not unduly biased by potential H isotope effects.

Groundwater depth emerged as a key environmental factor regulating plant water use strategies. *H. ammodendron* exhibited a clear shift from groundwater dependence under shallow conditions (GD_4.8_) to increased reliance on middle‐layer soil water (80–150 cm) as groundwater depth increased (GD_11.8_). The contribution of this layer rose from 12.3% to 43.2%, underscoring its importance under deeper groundwater conditions.

Distinct interspecific differences were observed along the gradient. Unlike *H. ammodendron*, *E. intermedia* , and *T. chinensis* , *C. mongolicum* consistently preferred shallow soil water (0–80 cm). However, this preference weakened at GD_9.4_, where water uptake became more evenly distributed across the 0–300 cm profile. The water use strategy of *T. chinensis* converged with that of *H. ammodendron*, with groundwater contribution up to 67.0%. In contrast, *E. intermedia* preferentially utilized deeper soil water, with its water uptake proportion increasing with soil depth.

Soil water and groundwater constituted the primary water sources for the studied species, whereas direct precipitation inputs were minimal (< 10%). Comparison of deuterium‐excess values (Figure 6a) revealed clear separation between precipitation and 0–80 cm soil water, which consistently exhibited strongly negative signatures across the groundwater gradient. The magnitude of this depletion exceeded precipitation variability, indicating substantial evaporative modification rather than simple rainfall infiltration. Nevertheless, pronounced interspecific differences persisted in the utilization of dominant sources and soil layers.

#### 3.3.2 Hydrological Niche Partitioning Among Coexisting Species

**FIGURE 7:**
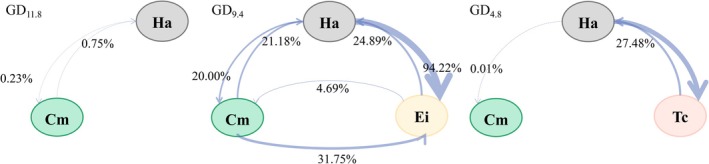
Overlapping node network diagram of hydrological niche of desert plants under different groundwater depths (GD_11.8_, GD_9.4_, GD_4.8_). A → B represents the probability of A being discovered within the hydrological niche area of B.

Niche overlap, a core metric for quantifying resource use similarity and interspecific relationships, was assessed using the nicheROVER model. The results revealed distinct patterns of hydrological niche overlap along the groundwater depth gradient. At the deepest depth (Figure 7), niche overlap between *H. ammodendron* and *C. mongolicum* was minimal (0.75% and 0.23%, respectively), indicating pronounced niche separation and highly divergent water use strategies. At the intermediate depth, a highly asymmetric overlap was observed between *E. intermedia* and *H. ammodendron*. The probability that *E. intermedia* 's niche region overlapped with that of *H. ammodendron* was 94.22%, suggesting *E. intermedia* 's high reliance on a water resource suite very similar to that of *H. ammodendron*. In contrast, *H. ammodendron*'s niche region overlapped with that of *E. intermedia* was only 24.89%, indicating that *H. ammodendron* utilized a broader range of resources beyond those shared with *E. intermedia* . Meanwhile, *C. mongolicum* maintained strong differentiation from all other species, showing the lowest overlap with *E. intermedia* (4.69%). At the shallowest depth, niche overlap became polarized. *T. chinensis* had a high overlap with *H. ammodendron* (68.07%), while conversely, the overlap is much lower (27.48%), indicating that *T. chinensis* 's hydrological niche is highly encompassed by *H. ammodendron*'s hydrological niche. And *C. mongolicum* showed complete or near zero overlap with all other species, reaffirming its unique hydrological niche.

## 4 Discussion

### 4.1 Study Limitations and Implications for Future Research

The interpretation of our findings should be considered in light of several constraints inherent to field‐based gradient studies. First, although the three sites were selected along a groundwater depth gradient, they also differ slightly in elevation (240–300 m). Elevational variation may covary with microclimatic conditions such as temperature and atmospheric evaporative demand, as well as soil physicochemical properties. Because these factors may partially covary with groundwater depth in the present observational framework, their independent effects cannot be fully separated. Consequently, part of the variation in vegetation composition and water use strategies may reflect combined hydrological and microclimatic influences, and the identified relationships should therefore be interpreted primarily as gradient associations rather than strict causal effects.

Second, the study included three groundwater levels representing typical community types in the region. While this design captures major contrasts in groundwater accessibility, it may not fully resolve fine‐scale ecological variation along a continuous hydrological gradient. Future studies incorporating a larger number of sampling sites under comparable topographic conditions would help refine the identification of ecological thresholds and improve the generality of the patterns reported here.

Third, sampling was conducted during the late growing season (August–September), when evaporative demand is typically high in the study region. Although this period facilitates the identification of relatively stable plant water use strategies (Chen and Zuo 2019), plant water source partitioning may vary across phenological stages and between years. Therefore, the strategies identified here should be regarded as seasonal expressions under high atmospheric demand rather than fixed year‐round traits. Multi‐season monitoring combined with measurements of soil salinity and root distribution would further improve understanding of the temporal stability of these mechanisms.

Despite these limitations, the present study provides field‐based evidence linking groundwater accessibility to vegetation structure and hydrological niche differentiation in a hyper‐arid desert ecotone.

### 4.2 Regulatory Mechanisms of Groundwater Fluctuations on Community Structure and Population Adaptation in Arid Plants

Groundwater depth fluctuations constitute a critical driver of plant community composition and spatial patterning in arid ecosystems (Elmore et al. 2006). The results suggest a dual‐threshold response along the groundwater gradient, where deep groundwater increases water limitation while shallow conditions may shift ecological constraints toward salinity and hypoxia (Huang et al. 2020). This framework explains the nonlinear species diversity pattern along groundwater depth gradients, where intermediate depths maximize species richness, whereas extreme conditions suppress biodiversity through distinct stress pathways.

For instance, the visual evidence of surface salt crusts at the shallowest groundwater depth (4.8 m) in this study points to substantial topsoil salt accumulation (Figure 1b). While direct physiological stress or soil salinity measurements were not conducted, recent studies in this region demonstrate that extreme evaporative demand can drive strong capillary rise, continuously transporting soluble salts to the root zone even from depths near 5 m (Lian et al. 2022; Wang et al. 2019). Therefore, plants at this site were likely subjected to persistent salinity stress. Notably, this 4.8 m depth is deeper than the critical threshold of 2.5 m for salt accumulation recorded in northern Fukang (Wang et al. 2008). This comparison suggests that the hydrological threshold for salt‐driven ecological degradation in this specific hyper‐arid ecotone may be deeper than that in other arid basins, exacerbating the constraints on plant survival.

At the species level, this study observed a unimodal distribution across groundwater depths of 4.8–11.8 m, contrasting with the continuous decline in population density reported by Mu et al. (2024). This discrepancy may stem from synergistic effects of salinity stress and interspecific competition in shallow groundwater environments. High salinity directly inhibited *H. ammodendron* recruitment, while the salt excreting species *T. chinensis* (Zhang, Qian, et al. 2024) outcompeted *H. ammodendron* through superior adaptation to saline conditions, further restricting population regeneration of *H. ammodendron*.

Meanwhile, the relative abundance of *C. mongolicum* (Cm) increased markedly in areas near the mountain front with greater groundwater depth, but gradually declined in regions with shallower groundwater depth. This distribution pattern appears closely linked to its water use strategy, which primarily relies on soil water content in the 0–80 cm layer. In mountain proximal zones, spring snowmelt provides a stable and sustained recharge to shallow soils, effectively meeting the water demands of *C. mongolicum* 's horizontally distributed root system. At the same time, greater groundwater depth reduces capillary rise and subsequent salt accumulation in surface soils, creating a more favorable water salinity environment that supports population expansion. By contrast, in areas farther from the mountains with shallower groundwater depth, shallow soil water is mainly recharged by sporadic rainfall, which is subject to high evaporative loss. Moreover, capillary upflow from shallow groundwater exacerbates surface salinity accumulation (Zhang, Lin, et al. 2024), thereby constraining the regeneration and survival of *C. mongolicum* populations.

At the population level, *H. ammodendron* shows a negative correlation between mean basal diameter and individual abundance (*r* = −0.68, *p* < 0.05), small diameter individuals dominate under shallow groundwater, while large diameter individuals become more prevalent under deep groundwater but with smaller canopies. This pattern reflects a classical trade off between survival and growth under water stress (Fantinato et al. 2023), in which plants allocate more resources to root development to access deeper water (Fan et al. 2017), at the expense of above ground growth, potentially resulting in carbon limitation under prolonged drought (Xu, McDowell, and Li 2016). Such resource allocation leads to stunted, aging populations with reduced reproductive potential, thereby limiting population renewal (Dai et al. 2022). Interestingly, *H. ammodendron* height does not exhibit a significant linear relationship with groundwater depth, consistent with Xu et al. (2017), indicating that plant responses to groundwater fluctuations are mediated by complex ecological processes rather than simple linear dynamics.

### 4.3 Species Specific Water Use Strategies Driven by Groundwater Fluctuations

Building on community level responses, species specific water use strategies elucidate the mechanisms through which groundwater fluctuations shape community dynamics. In this hyper‐arid region, precipitation contributes little to plant water supply, as its low frequency and intensity result in negligible soil moisture recharge (Li et al. 2013), and high evaporative demand rapidly removes infiltrated water (Bowles et al. 2018). Groundwater in the study area is mainly recharged by precipitation and snowmelt originating from the Tianshan Mountains, which is transported to the desert plain through river infiltration and subsurface flow. Consequently, groundwater and deep soil water represent the dominant and more reliable sources sustaining vegetation. The relatively depleted isotopic signatures observed in deeper soil layers also suggest strong hydraulic connectivity with groundwater through capillary rise rather than direct infiltration of local rainfall.

As groundwater depth increased, *H. ammodendron* exhibited a pronounced shift in water sourcing, transitioning from shallow groundwater (> 50%) to unsaturated zone soil water. Given the isotopic similarity between deep soil water and groundwater, contributions from groundwater may be slightly overestimated, suggesting stronger reliance on deep soil reserves. This flexible response contrasts with obligate phreatophytes (Torres‐García et al. 2021), indicating that *H. ammodendron* functions as a facultative groundwater user capable of reallocating water uptake as accessibility changes. However, once groundwater falls beyond rooting limits, dependence on unsaturated zone water alone may constrain regeneration and long‐term carbon balance (Zhao et al. 2021).

In contrast, *C. mongolicum* relies mainly on shallow to intermediate layer soil water (0–150 cm) (Le Houérou 1996; Xu, Ji, et al. 2016). At the GD_9.4_ site, *C. mongolicum* (Cm) exhibited significant plasticity by expanding its water uptake uniformly across the 0–300 cm soil profile. However, soil water profiles did not show clear depletion signals in these shallow layers. This implies that *C. mongolicum* 's downward expansion of its hydrological niche is not a forced response to shallow water scarcity (resource limitation), but rather an adjustment primarily driven by interspecific competition—likely expanding its root functional zone to minimize niche overlap with coexisting species (Antunes et al. 2019).

*E. intermedia* exhibits strong reliance on both deep soil water and groundwater (Hasselquist and Allen 2009; Wang et al. 2017). *T. chinensis* shows the highest groundwater utilization (67%), consistent with regional studies (Dong et al. 2024; Xu et al. 2024). Its dual layer root system confers high plasticity in water use and competitive dominance under shallow, high salinity conditions, where it can impose exclusionary effects on *H. ammodendron* (Dong et al. 2024). Although dimorphic root systems may facilitate hydraulic redistribution in desert shrubs (Dawson and Pate 1996), the isotopic profiles observed in this study did not show clear evidence of such processes. Hydraulic redistribution typically occurs at diel timescales and depends on transient soil moisture gradients between soil layers, which may not be detectable from single‐time sampling of soil water isotopes. Therefore, while the studied species may potentially perform hydraulic redistribution, our dataset does not provide direct evidence for this mechanism.

Overall, groundwater depth selects for a suite of distinct water use strategies, ranging from the deep groundwater specialization of *T. chinensis* and *E. intermedia* to the shallow soil foraging of *C. mongolicum* , with *H. ammodendron* exhibiting high plasticity in between. These divergent strategies form the mechanistic basis for species interactions.

### 4.4 Response Mechanisms of Hydrological Niche Overlap and Separation to Groundwater Depth

Understanding plant water use strategies and the resulting hydrological niches is fundamental for interpreting species coexistence in water‐limited systems (Silvertown et al. 2015). The degree of niche overlap reflects similarity in resource use and environmental adaptation (Zhao, Wang, et al. 2024). Previous studies have shown that under water stress, coexisting species may reduce direct competition through stratified water uptake, thereby promoting community stability (Wu et al. 2022). However, such coexistence patterns are constrained by morphological and physiological trade‐offs that determine how species partition limited resources (Muñoz‐Gálvez et al. 2025).

At the deepest groundwater depth (11.8 m), overlap between *H. ammodendron* and *C. mongolicum* was extremely low (< 1%), coinciding with the lowest plant density. This pattern is consistent with the interpretation that strong drought stress associated with limited groundwater accessibility reduces community carrying capacity, allowing persistence primarily by species capable of deep water acquisition. Under such conditions, coexistence appears to be associated with pronounced resource partitioning and minimal niche overlap (McCutcheon et al. 2017). Similar hydrological niche segregation has been widely reported across arid ecosystems, including extreme arid regions of Northwest China (Fan et al. 2025; Gu et al. 2025; Zhou et al. 2025), desert steppes (Song et al. 2025), and gypsum‐calcareous systems (Rudov et al. 2023), where spatiotemporal partitioning of water resources is commonly observed. By developing contrasting water use strategies, groundwater‐dependent shrubs may reduce interspecific competition under severe hydrological deficits (Liu et al. 2024). Within this broader context, our results suggest that declining groundwater accessibility is associated with increased niche differentiation.

At intermediate groundwater depth (9.4 m), species richness peaked, indicating that moderately accessible water likely alleviated environmental stress and permitted the establishment of more water‐sensitive species. At this depth, *E. intermedia* and *H. ammodendron* exhibited extremely high asymmetric niche overlap (94.22%). Although high isotopic overlap can imply potential strong competition, coexistence under these conditions may be facilitated by relatively relaxed water limitation and differentiation along other functional axes (Amarasekare 2003). Root architectural differences and physiological traits including the shallow horizontal roots of *C. mongolicum* and the deeper taproots of *H. ammodendron* and *T. chinensis* may buffer direct competitive exclusion even when isotopic signatures are similar, consistent with coordination between rooting depth and plant ecological strategies reported in dryland systems (Illuminati et al. 2022). Moreover, the salt‐excreting mechanism of *T. chinensis* enhances tolerance to salinity that may constrain *H. ammodendron*. Thus, apparent isotopic overlap does not necessarily indicate identical ecological niches but may reflect shared access to a bulk water pool combined with differentiation in morphology or stress tolerance (Letten et al. 2018; Pastore et al. 2021).

However, this state of high overlap coexistence appears sensitive to hydrological shifts (Hastings 2004). At the shallowest groundwater depth (4.8 m), species richness declined while plant density increased. The reduction in *E. intermedia* abundance and concurrent rise of *T. chinensis* suggest that secondary stresses, particularly salinity, may restructure competitive hierarchies. Niche overlap analysis revealed polarized interactions, including asymmetric overlap between *T. chinensis* and *H. ammodendron*, while *C. mongolicum* maintained minimal overlap yet stable relative abundance (12.73%). This pattern indicates that under conditions of relatively high resource availability but intensified stress and competition, some species maintain persistence through strong niche differentiation (McCutcheon et al. 2017; Silvertown et al. 1999), whereas others experience competitive displacement.

Overall, the results indicate a nonlinear association between groundwater depth and hydrological niche structure. Deep groundwater levels correspond with low‐overlap coexistence under drought stress; intermediate depths permit high isotopic overlap potentially buffered by trait differentiation; and shallow levels combine water accessibility with salinity stress, leading to reorganization of competitive relationships. While further validation across seasons and replicated gradients is required, these findings contribute to a process‐based understanding of how groundwater variability shapes species interactions in hyper‐arid ecosystems.

## 5 Conclusions

This study elucidates the nonlinear ecohydrological patterns associated with plant community assembly and water use strategies along groundwater depth gradients in the hyper‐arid Junggar Basin, highlighting three principal contributions. First, our results reveal a pattern consistent with a “dual‐threshold” hydrological filter regulating biodiversity, with species richness peaking at intermediate groundwater depths, while declining under shallow conditions potentially constrained by salinity‐related stress (< 5 m) and under deep conditions characterized by hydraulic limitation (> 12 m). Although based on discrete gradient points, this nonlinear response suggests the presence of hydrological constraints at both extremes of groundwater accessibility. Second, we observed contrasting coexistence strategies among dominant species. The broad hydrological niche of *H. ammodendron* appears associated with flexible water‐source utilization, including shifts toward deeper soil water under increasing stress, whereas *C. mongolicum* maintained relatively low niche overlap with coexisting species, indicating a conservative and competitively avoiding strategy. These patterns are consistent with divergent adaptive strategies facilitating persistence along environmental gradients. Third, niche dynamics exhibited clear state dependence. Under deep groundwater conditions, low niche overlap indicated pronounced resource partitioning, whereas at intermediate depths, high overlap emerged under relatively alleviated water limitation. At shallow depths, changes in species dominance and overlap patterns suggest that secondary stress factors may restructure competitive relationships. Based on these findings, we propose that maintaining groundwater levels within an intermediate range (approximately 5–10 m in the present system) may help mitigate risks associated with both drought‐induced vegetation decline and shallow water‐related degradation. In restoration practice, species exhibiting broader hydrological flexibility, such as *H. ammodendron*, may be more suitable for areas with deeper or highly fluctuating groundwater tables, whereas niche‐specialized species like *C. mongolicum* may contribute to stabilizing communities under intermediate hydrological conditions. Together, these results suggest that groundwater depth regulates species coexistence in desert ecotones by simultaneously shaping water availability, salinity stress, and hydrological niche partitioning.

## Author Contributions

**Meifei Zhu:** conceptualization (lead), data curation (lead), methodology (lead), visualization (lead), writing – original draft (lead). **Youyan Zhang:** funding acquisition (lead), project administration (lead), writing – review and editing (lead). **Xianglian Wang:** investigation (equal). **Jinhua Cheng:** writing – review and editing (lead). **Chunying Lei:** investigation (equal). **Zhengwei Han:** investigation (equal).

## Funding

This work was supported by National Key Research and Development Project of China, 2022YFF1302504 and 202402012‐2036.

## Conflicts of Interest

The authors declare no conflicts of interest.

## Supporting information


**Figure S1:** Comparison of single isotope and double isotope results.

## Data Availability

The datasets generated during the current study have been depositedare available in National Ecosystem Data Bank (https://ecodb.scidb.cn/). DOI: https://www.doi.org/10.57760/sciencedb.ecodb.00223.
