# Supplementary material for: Groundwater Controls on Plant Community Structure and Species Specific Water Use Strategies in the Desert Oasis Ecotone of the Junggar Basin
Source: Ecol Evol. 2026 Apr 7;16(4):e73443. doi: 10.1002/ece3.73443 (PMC13054237; doi:10.1002/ece3.73443)
Supplement: Supplementary file 1 — Figure S1: Comparison of single isotope and double isotope results. [file ECE3-16-e73443-s001.docx]

**Groundwater controls on plant community structure and species specific water use strategies in the desert oasis ecotone of the Junggar Basin**

Meifei Zhu^a^, Youyan Zhang^b,^*, Xianglian Wang^a^, Jinhua Cheng^a,^*, Chunying Lei^c^, Zhengwei Han^c^

*^a^College of Soil and Water Conservation, Beijing Forestry University, Beijing 100083, China*

*^b^Institute of Ecological Protection and Restoration, Chinese Academy of Forestry Sciences, Beijing 100091, China*

*^c^Xinjiang* *Jinghe Desert Ecosystem National Positional Observatory,* *Institute of Silviculture and Sand Control, Xinjiang Forestry Academy of Sciences, Urumqi 830063, China*

*Corresponding authors.

*Email address:* [youyanzh@caf.ac.cn](mailto:youyanzh@caf.ac.cn); [Jinhua_cheng@126.com](mailto:Jinhua_cheng@126.com)

**Supplementary Materials**

| 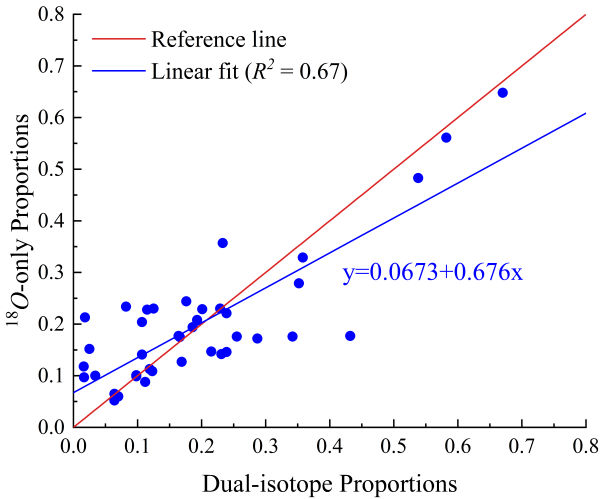 |
| --- |
| **Figure S1** Comparison of single isotope and double isotope results. |
